# Emerging food- and waterborne pathogen *Arcobacter* in wastewater: diversity and antibiotic resistance

**DOI:** 10.1128/spectrum.03799-25

**Published:** 2026-09-17

**Authors:** Svjetlana Dekić Rozman, Antti Karkman, Levi Tomitšik, Marko Virta

**Affiliations:** 1 Department of Microbiology, University of Helsinki 3835, Helsinki, Finland; University of Kentucky, Lexington, Kentucky, USA

**Keywords:** *Arcobacter*, wastewater treatment plant, antibiotic resistance, full-length 16S rRNA sequencing, whole-genome sequencing, sewage

## Abstract

*Arcobacter* spp. are emerging food- and waterborne pathogens frequently detected in wastewater. Despite their high abundance in wastewater, *Arcobacter* diversity, antibiotic resistance, and genomic traits remain poorly characterized. To address these knowledge gaps, we conducted a comprehensive study of *Arcobacter* spp. in influent, effluent, and activated sludge from a Finnish wastewater treatment plant using full-length 16S rRNA gene sequencing, isolate-based genomics, and phenotypic antibiotic susceptibility testing. *Arcobacter* spp. were highly abundant in raw sewage but substantially removed during treatment. Four *Arcobacter* species were identified, dominated by *Arcobacter cryaerophilus* and *Arcobacter suis*. A proportion of amplicon sequence variants unclassified to species-level revealed potentially unexplored *Arcobacter* diversity. For the first time, we observed intragenomic variability in 16S rRNA gene copies of *A. cryaerophilus*, highlighting the importance of integrating culture-based and culture-independent approaches. Phenotypic testing revealed high proportions of non-wild-type isolates for clinically relevant antibiotics, including ampicillin, cefotaxime, tetracycline, and erythromycin. Genomic analyses showed that antibiotic resistance profiles were primarily mediated by chromosomally encoded determinants, including β-lactamases, efflux systems, and point mutations. Additionally, a broad arsenal of chromosomal and plasmid-borne resistance genes to heavy metals, biocides, and organic solvents was detected, reflecting adaptations to the wastewater environment. These findings provide novel insights into *Arcobacter* species-level diversity, resistance mechanisms, and ecological adaptations in anthropogenically influenced environments. The study highlights the significance of *Arcobacter* for public health and establishes a foundation for further research.

**IMPORTANCE:**

*Arcobacter* spp. are emerging human and animal pathogens that exhibit increasing resistance to clinically relevant antibiotics. Most community-acquired infections are linked to exposure through contaminated food and water, yet studies investigating their occurrence and diversity in wastewater remain scarce. Here, we focus on wastewater as an abundant source of *Arcobacter* spp. and a potential dissemination route contributing to downstream contamination of surface waters, irrigated soils, and possibly the food chain. By characterizing the species-level diversity, genomic traits, and antibiotic resistance profiles of *Arcobacter* spp. in wastewater, this study provides critical insights into the ecology and epidemiology of this ubiquitous genus.

## INTRODUCTION

Bacteria belonging to the *Arcobacter* genus are gram-negative, non-spore-forming, motile curved rods capable of growing under both microaerobic and aerobic conditions. Initially classified as *Campylobacter*, these bacteria were redefined as a separate genus by Vandamme et al. (1). Taxonomy within this group has undergone major revisions in recent years, with some researchers subdividing the genus *Arcobacter* into seven distinct genera (2) and others insisting on keeping *Arcobacter* as a single distinct genus (3, 4). The International Committee on Systematics of Prokaryotes reached a consensus to keep *Arcobacter* as a single genus (5). However, some genomic databases still follow the split-genus classification, leading to inconsistent reports in literature. In the present study, the genus name *Arcobacter* was used throughout to maintain consistency with the current taxonomic consensus recognizing *Arcobacter* as a single genus (5).

Currently, 37 described species are within the *Arcobacter* genus (LPSN [6]). Among them, *Arcobacter butzleri*, *Arcobacter cryaerophilus*, and *Arcobacter skirowii* are clinically significant, causing diarrhea, gastroenteritis, bacteremia, endocarditis, and peritonitis in humans, as well as mastitis, abortions, and diarrhea in animals (7, 8). Furthermore, A. *butzleri* and *A. cryaerophilus* are categorized as foodborne pathogens by the International Commission on Microbiological Specification for Food (9). Community-acquired *Arcobacter* infections are linked with exposure to water contaminated through sewage as well as the consumption of food products (vegetables, dairy products, chicken, and pork meat) that can be contaminated during any stage of the food chain (10).

*Arcobacter* spp. are prevalent in diverse aquatic environments, including groundwater, spring water, drinking water, rivers, seawater, and canals, and have the highest prevalence in sewage, highlighting their potential as waterborne pathogens (11, 12). Their prevalence in freshwater and seawater correlates with high levels of fecal pollution (13). Wastewater treatment plants (WWTPs) are of particular concern, as *Arcobacter* spp. show variable removal rates during treatment processes, potentially allowing them to persist and spread downstream (11, 12). Recent studies further suggest that *Arcobacter* spp. may act as hosts for clinically significant antibiotic resistance genes in wastewater (14–16). Using epicPCR (emulsion paired isolation and concatenation PCR), studies identified *Arcobacter* as a carrier of β-lactam, tetracycline, and biocide resistance genes, as well as class 1 integrons (14–16).

Antimicrobial resistance in *Arcobacter* spp. is an emerging concern, with increasing reports of resistance to fluoroquinolones, macrolides, tetracyclines, and aminoglycosides, which are antibiotics critical for the treatment of human infections (17, 18). Despite this, standardized guidelines for antibiotic susceptibility testing (AST) are lacking, making inter-study comparisons challenging (17, 18).

*Arcobacter* spp. are increasingly recognized as emerging enteric pathogens and are frequently detected in wastewater, yet their species-level diversity, resistance determinants, and survival strategies in WWTPs remain poorly characterized. This knowledge gap is critical because both treated and untreated wastewater, as well as sewage sludge, can facilitate the environmental dissemination of *Arcobacter*, contributing to downstream contamination of surface waters and agricultural soils, and potentially entering the food chain through the irrigation of fresh produce and the exposure of food-producing animals to contaminated water or soil. This study represents a systematic investigation of *Arcobacter* diversity, antibiotic resistance, and genetic traits in a Finnish WWTP using amplicon sequencing and isolate-based genomic and phenotypic antibiotic susceptibility testing.

## MATERIALS AND METHODS

### Sampling

Samples of raw wastewater (influent), activated sludge, and treated wastewater (effluent) were collected in November 2023 from Viikinmäki WWTP in Helsinki, Finland. This WWTP has a capacity for 800,000 residents and receives approximately 15% industrial and 85% municipal wastewater (15). Wastewater was obtained as 24-h composites, while activated sludge was collected at a single time point. Immediately after collection, the samples were transferred to the laboratory and processed.

### Isolation of *Arcobacter* spp.

*Arcobacter* isolates were cultivated on *Arcobacter* agar (*Arcobacter* broth, Oxoid, UK, and 12 g/L agar, Thermo Scientific, USA) with CAT supplement (Oxoid) containing cefoperazone (8 µg/mL; third-generation cephalosporin, inhibition of most gram-negative bacteria), amphotericin B (10 µg/mL, antifungal), and teicoplanin (4 µg/mL, inhibiting gram-positive bacteria). To increase the selection pressure toward resistant isolates, cultivation was also performed on *Arcobacter* agar supplemented with CAT and tetracycline (2 µg/mL, 98% purity; Merck, USA), hereafter referred to as CTET. Cultivation was carried out both with and without enrichment in *Arcobacter* CAT broth (30°C, 48 h, aerobic conditions). All agar plates were incubated aerobically at 30°C for 48 h to avoid culturing *Campylobacter* spp. that favor microaerobic conditions.

For influent and effluent samples, 10 mL of influent and 100 mL of effluent were filtered in duplicate through 0.2 µm membrane filters (ME 24/21 ST; Whatman, UK). One filter from each sample was placed in 9 mL *Arcobacter* CAT broth, vortexed to dislodge bacteria, and serially diluted (1 mL into 9 mL of 0.9% NaCl) up to 10⁻⁴, and 100 µL of each dilution was plated on *Arcobacter* CAT and CTET agar. The duplicate filter was enriched in 9 mL *Arcobacter* CAT broth (30°C, 48 h). Since *Arcobacter* spp. can pass through small pores, the enrichment broth, after incubation, was vortexed and filtered through a 0.45 µm syringe filter (PES, Millex; Merck) to reduce the growth of background bacteria. The filtrate was serially diluted to 10⁻², plated on *Arcobacter* CAT and CTET agar, and incubated (30°C, 48 h).

For activated sludge, 10 mL was added to 90 mL *Arcobacter* CAT broth, mixed, serially diluted to 10⁻⁴, and plated as above. To further enrich, the same broth was incubated at 30°C for 48 h, filtered through 0.45 µm PES syringe filters (several filters were required due to clogging), serially diluted to 10⁻⁴, plated on *Arcobacter* CAT and CTET agar, and incubated. Presumptive *Arcobacter* spp. colonies were subcultured, purified, and stored in 30% glycerol at −80°C. Identification of the isolates was performed with matrix-assisted laser desorption/ionization time-of-flight mass spectrometry (MALDI Biotyper; Bruker, Germany).

### Antibiotic susceptibility testing

Antibiotic susceptibility testing of 81 recovered *Arcobacter* isolates for six clinically relevant antibiotics—ampicillin, cefotaxime, ciprofloxacin, erythromycin, gentamicin, and tetracycline—was performed using minimum inhibitory concentration (MIC) gradient strips (Liofilchem, Italy). The selected antibiotics represent different antibiotic classes and are of significant clinical relevance.

Testing was conducted according to modified European Committee on Antimicrobial Susceptibility Testing (EUCAST) guidelines for *Campylobacter. Arcobacter* isolates were grown on *Arcobacter* agar plates at 30°C for 48 h in aerobic conditions. Bacterial biomass was collected with a cell scraper, suspended in 0.9% NaCl, and adjusted to 0.5 McFarland. Using a cotton swab, the suspension was spread on Mueller–Hinton agar with 5% defibrinated horse blood and NAD (Thermo Scientific) to create a bacterial lawn. After the inoculated plates dried, the antibiotic strip was placed in the center. Plates were incubated at 30°C for 48 h in chambers with CampyGen sachets (Oxoid) to generate microaerobic conditions supporting optimal growth. Antimicrobial susceptibility testing was performed as a single measurement per isolate, with repeat testing conducted only when results were ambiguous or technically uncertain. This approach reflects the absence of standardized AST protocols for *Arcobacter* spp. and introduces a limitation regarding the reproducibility of MIC values, with repeated testing for ambiguous or borderline results. Quality control was done with *Escherichia coli* DSM 1103 in aerobic conditions at 37°C for 24 h. All quality control results were within the expected acceptable ranges under the standard testing conditions, supporting the validity of the assay performance.

In the absence of clinical breakpoints or epidemiological cutoff (ECOFF) values for *Arcobacter* spp., ECOFF values for *Campylobacter jejuni* were used to interpret susceptibility profiles (19): ampicillin, 16 µg/mL; ciprofloxacin, 0.5 µg/mL; erythromycin, 4 µg/mL; gentamicin, 2 µg/mL; tetracycline, 1 µg/mL. The interpretation of the results is consistent with previous studies (17, 20, 21). Isolates with MIC values above the designated ECOFF were classified as non-wild type (potential acquired resistance mechanisms), whereas isolates with MIC values equal to or below the ECOFF were considered wild type (no acquired resistance mechanisms). Since no official ECOFF or clinical breakpoint is available for cefotaxime for *Arcobacter* spp., a provisional threshold of 16 µg/mL was applied for descriptive visualization of MIC distributions. This value was selected based on related β-lactam antibiotics within the same class for *Campylobacter* and used solely for exploratory data presentation; it does not represent a validated interpretive breakpoint.

### DNA extraction

For genomic DNA extraction, *Arcobacter* isolates were grown in 5 mL *Arcobacter* broth at 30°C for 48 h. Several *A. cryaerophilus* isolates that yielded insufficient biomass in broth were instead cultivated on multiple *Arcobacter* agar plates, and the resulting biomass was pooled prior to extraction. Genomic DNA was extracted using the DNeasy Blood & Tissue Kit (QIAGEN, USA) following the manufacturer’s protocol for gram-negative bacteria. DNA concentration and purity were assessed with a NanoDrop One spectrophotometer (Thermo Scientific).

For total environmental DNA, 20 mL of influent and 200 mL of effluent were filtered through 0.22 µm membrane filters (ME 24/21 ST, Whatman) in triplicate. DNA was extracted from filters using the DNeasy PowerWater Kit (QIAGEN). From 0.5 mL of settled activated sludge, DNA was extracted in triplicate using the DNeasy PowerLyzer PowerSoil Kit (QIAGEN).

### Sequencing

The full-length 16S rRNA gene was amplified from total environmental DNA using the universal primer set AGRGTTYGATYMTGGCTCAG (27F) and RGYTACCTTGTTACGACTT (1492R). For whole-genome sequencing, 33 isolates were selected based on their antibiotic resistance profiles, prioritizing those with higher MIC values. The selected isolates originated from influent wastewater (*n* = 11), effluent wastewater (*n* = 13), and activated sludge (*n* = 9). Most *A. butzleri* isolates (27/33) were obtained from enriched samples, whereas *A. cryaerophilus* was recovered only from non-enriched samples. Therefore, all six *A. cryaerophilus* isolates were included. This selection strategy ensured representation of both species and their resistance diversity across the sampled matrices. Both 16S rRNA amplicons and genomic DNA were sequenced with PacBio Revio (Pacific Biosciences, USA) at the Institute of Biotechnology, University of Helsinki, Finland to obtain long HiFi reads.

### 16S rRNA gene amplicon analysis

Primer trimming and read orientation were performed with Cutadapt (v4.9) (22). Read quality was assessed with FastQC (v0.11.9) (23) and summarized with MultiQC (v1.14) (24). Amplicon sequence variant (ASV) inference was performed in R (v4.4.2) (25), using the DADA2 (v1.34.0) pipeline (26). Taxonomic annotation was performed against the SILVA database (v138.1) (27), and bacterial community composition was analyzed using the phyloseq package (v1.50.0) (28). Principal coordinate analysis (PCoA) ordination was constructed based on Aitchison distances and visualized with microViz (v0.12.7) (29).

### Whole-genome analysis

The 33 genomes were assembled using the Autocycler (v0.2.0) workflow (30), which generates and reconciles multiple independent assemblies produced by Flye (v2.9.5-b1801) (31), Canu (v2.2) (32), miniasm (33), Raven (v1.8.3) (34), and LJA (35) to obtain consensus genome assemblies.

The completeness and contamination of isolates were checked with CheckM2 (v1.0.1) (36), and taxonomic annotation was performed with GTDB-Tk (v2.4.0) (37) against the Genome Taxonomy Database release 220 (38). Genome annotation was done with Bakta (v1.9.2) using the full database (v5.1) (39). Resistance genes were annotated with ABRicate (v1.0.1) (40) against ResFinder (41), CARD (42), PlasmidFinder (43), and Bacmet (44) databases, applying the default thresholds of 80% nucleotide sequence identity and 80% sequence coverage. These parameters were used to balance sensitivity and specificity and to reduce the inclusion of false-positive hits from closely related non-resistance genes. Resistance mutations in the gyrA gene were annotated manually. Genetic contexts of resistance genes were inspected by extracting the gene and its flanking regions from Bakta-annotated GenBank files and visualized with clinker (v0.0.31) (45).

A plasmid gene-sharing network was constructed based on pairwise DNA sequence similarity of 90% using Biopython (v1.80) in Python (v3.10.6). Visualization was done with matplotlib (v3.6.2) in Python (v3.10.6).

### Phylogenetic analysis

Phylogenetic analysis of the 16S rRNA gene was done by downloading aligned reference sequences from the Silva database (27). All full-length amplicon ASVs assigned to *Arcobacter* and unique 16S rRNA sequences from each isolate were added to the reference alignment with MAFFT (v7.525) (46). The phylogenetic tree was created with RAxML (v8.2.12) (47) using the GTRGAMMA substitution model with the *Campylobacter* 16S rRNA gene sequences as an outgroup.

For whole-genome phylogenetic analysis, shared single-copy core genes (SCGs) were identified using anvi’o (v8) (48) pangenomics workflow (49). The SCGs were aligned with MUSCLE (50) within anvi’o (v8), and alignment was trimmed with trimAl (v1.4.rev22) (51). The phylogenetic tree was constructed with FastTree (52) within anvi’o (v8).

### Statistical analysis

All statistical analyses were conducted in R (v4.4.2) (25). Data handling and visualization were performed with the tidyverse suite (v2.0.0) (53), ggplot2 (v3.5.2) (54), and ComplexHeatmap (v2.22.0) (55). Phylogenetic tree implementation into figures was conducted using ape (v5.8.1) (56), and sequence handling was performed with Biostrings (v2.76.0) (57). For visualization of antibiotic resistance profiles, a heatmap was generated by dividing MIC values of individual antibiotics by the corresponding ECOFF value and applying a log_2_ transformation.

Multivariate analyses of log₂-transformed MIC values were performed using vegan (v2.8.0) (58). Permutational multivariate analysis of variance (PERMANOVA) was conducted with adonis2() to test the effects of species and isolation source on MIC profiles. PCoA was performed to visualize relationships among isolates with classical multidimensional scaling [cmdscale()] on Euclidean distances.

## RESULTS

### Bacterial community composition

**Fig 1:**
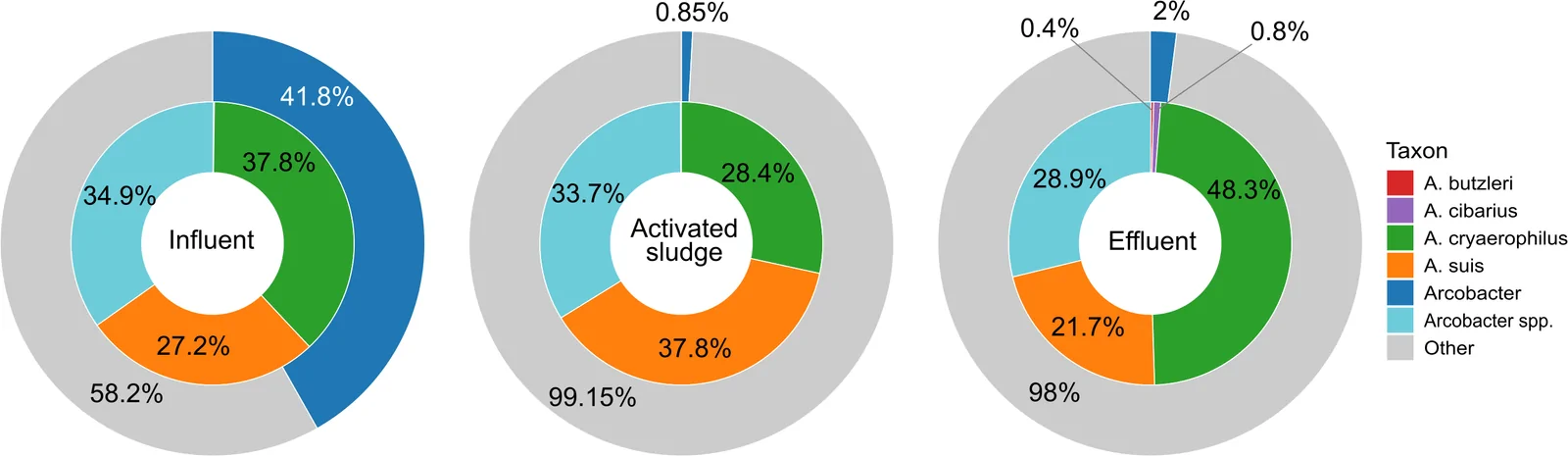
Relative abundance of *Arcobacter* and other bacterial genera in influent, activated sludge, and effluent samples from WWTP Viikinmäki is shown in the outer ring. The inner ring shows the species-level composition of *Arcobacter* sequences. *A. cryaerophilus* and *A. suis* represented the most abundant classified species, while the light blue (*Arcobacter* spp.) segment indicates *Arcobacter* sequences that could not be classified to the species level.

Amplicon sequencing analysis revealed that the relative abundance of the *Arcobacter* genus was highest in raw sewage (41.8%), followed by 1.9% in effluent, and 0.8% in activated sludge (Fig. 1). The overall bacterial community composition was different between influent, activated sludge, and effluent samples. The 20 most abundant genera are presented in Fig. S1.

In total, 225 distinct ASVs were assigned to *Arcobacter*. Most belonged to four species: *A. cryaerophilus* (73 ASVs), *Arcobacter suis* (51 ASVs), *A. cibarius* (3 ASVs), and *A. butzleri* (2 ASVs). Furthermore, 96 ASVs could not be confidently assigned to any *Arcobacter* species.

Species-level proportions within the *Arcobacter* genus varied across treatment stages. The proportion of *A. cryaerophilus* was lower in activated sludge (28.4%) compared to influent (37.8%) and higher in effluent (48.3%), where it became the dominant *Arcobacter* species. *A. suis* showed a higher proportion in activated sludge (37.8%) than in influent (27.2%) and a lower proportion in effluent (21.7%). *A. butzleri* and *A. cibarius* were rare in influent (0.1%) and showed slightly higher proportions in effluent (0.4% and 0.8%, respectively), while they were not detected in activated sludge (Fig. 1).

### *Arcobacter* cultivation and antibiotic resistance profile

In total, 81 *Arcobacter* isolates were recovered, comprising 75 *A. butzleri* (influent = 24, activated sludge = 24, and effluent = 27) and 6 *A. cryaerophilus* (influent = 2 and effluent = 4). Despite culturing on selective plates containing antibiotics, substantial background bacterial growth was observed. Syringe filtration of the incubated enrichment broth successfully reduced this background growth. The enrichment step predominantly favored the isolation of *A. butzleri*, whereas *A. cryaerophilus* was recovered only from non-enriched samples through careful plate examination.

**Fig 2:**
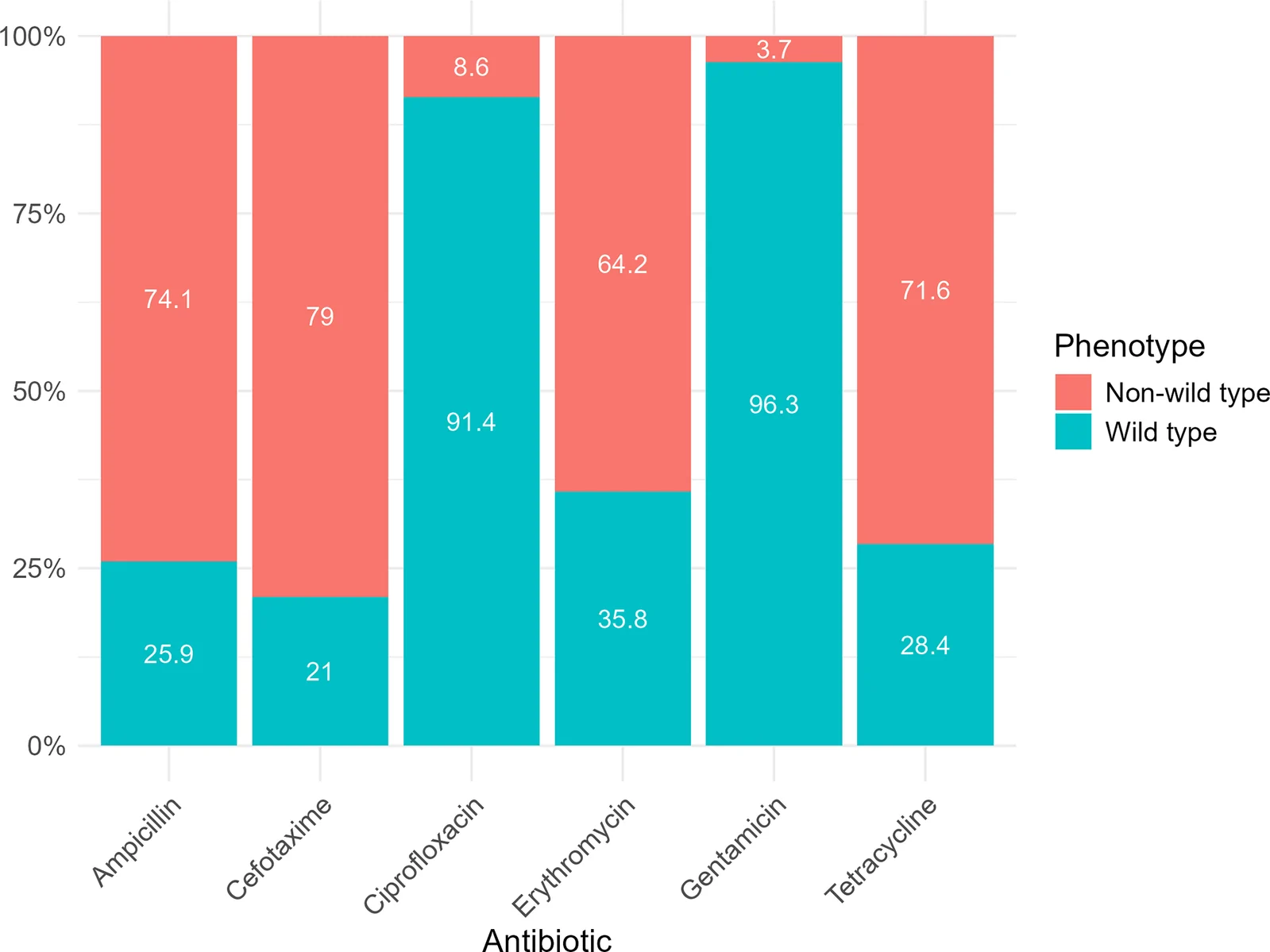
Percentage of wild and non-wild phenotypes among *Arcobacter* isolates (*n* = 81) for ampicillin, cefotaxime, ciprofloxacin, erythromycin, gentamicin, and tetracycline. The phenotype was determined by using *Campylobacter jejuni* epidemiological cutoff (ECOFF) values. Since no official ECOFF value for cefotaxime is available for *C. jejuni*, a hypothetical threshold value of 16 μg/mL was applied for visualization and statistical analyses.

The antibiotic resistance profiles of all isolates are presented in Table S1. The highest MIC values were observed for ampicillin and cefotaxime, with values exceeding 256 µg/mL in some isolates. A non-wild phenotype was most frequently detected for cefotaxime (79%), ampicillin (74.1%), tetracycline (71.6%), and erythromycin (64.2%). In contrast, only 8.6% and 3.7% exhibited a non-wild phenotype for ciprofloxacin and gentamicin, respectively (Fig. 2).

Selective culturing influenced the resistance profiles of the recovered isolates. Plates containing only CAT supplement yielded 45 isolates with mixed phenotypes and 11 isolates with a wild phenotype for all tested antibiotics. In comparison, selective culturing on plates containing tetracycline (CTET) yielded 25 isolates with a non-wild phenotype for ampicillin, cefotaxime, tetracycline, and erythromycin (only five isolates had MIC below the ECOFF for erythromycin), suggesting that the addition of tetracycline facilitated the recovery of isolates with non-wild phenotypes for multiple antibiotics (Table S1).

Antibiotic resistance profiles were compared between isolates to determine whether the isolation source (influent, activated sludge, and effluent) or species (*A. butzleri*, *n* = 75; *A. cryaerophilus*, *n* = 6) influenced observed MIC values (Fig. S2). PERMANOVA showed no significant effect of the isolation source, but a significant species effect was detected (*R*² = 8.4%, *P* = 0.02), indicating that species explain a small, yet statistically detectable, portion of the total variation in MIC profiles. However, the strong imbalance in sample sizes limits confidence, and additional data for *A. cryaerophilus* would strengthen these conclusions.

### Genomics

For whole-genome sequencing, 33 isolates (*A. butzleri*, *n* = 27; *A. cryaerophilus*, *n* = 6) were selected based on their antibiotic resistance profile. The criteria for selection were having MICs for individual antibiotics well above and below the ECOFF threshold to be able to distinguish the genetic mechanisms underlying the observed antibiotic resistance phenotypes.

**Fig 3:**
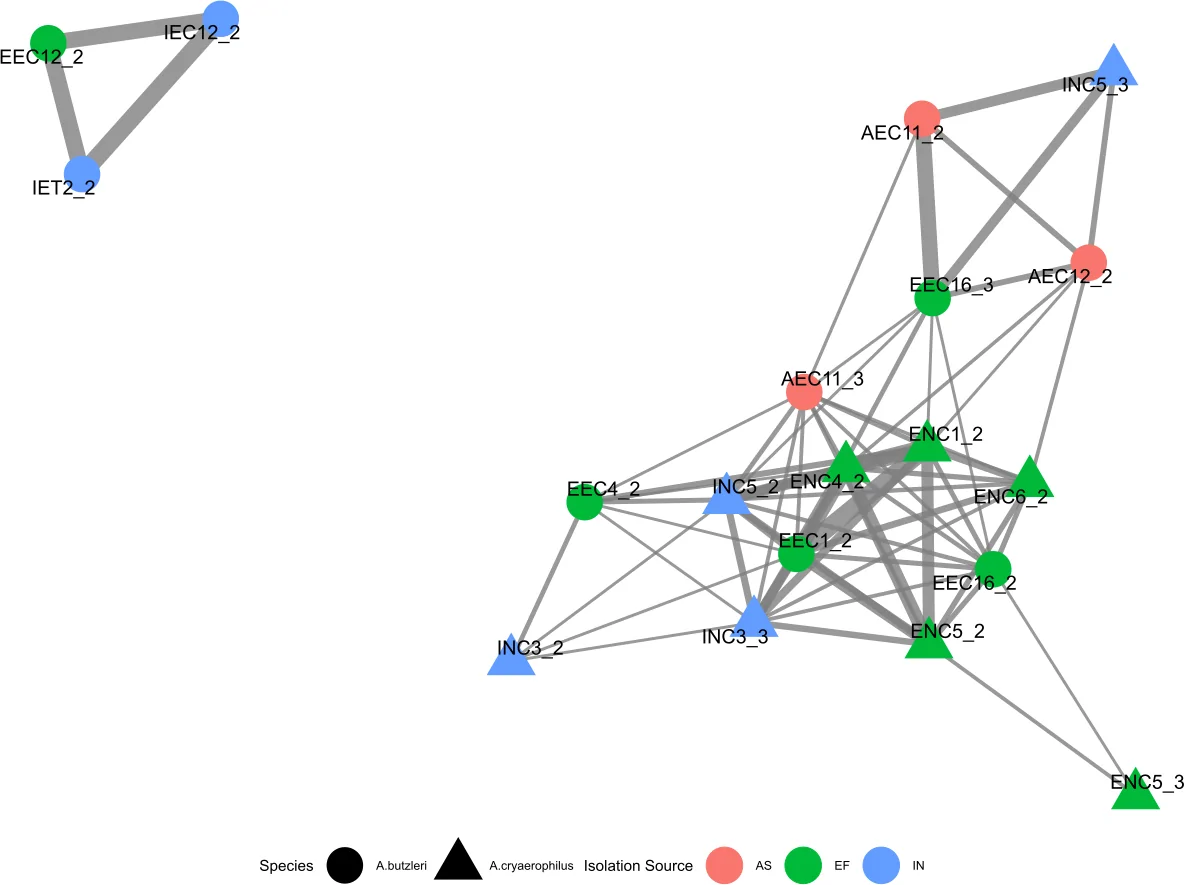
Plasmid gene sharing network between the plasmids within isolates. The width of the edge corresponds to the number of shared genes. Only genes with similarity above 90% are considered. Plasmids from *A. butzleri* are denoted with circles and from *A. cryaerophilus* with triangles. Nodes are named based on the isolate code and replicon number to differentiate between different plasmids from the same isolate. Colors represent the isolation source. AS, activated sludge; EF, effluent; IN, influent.

Genetic characteristics of the sequenced isolates are presented in Tables S2 and S3. The average genome size of both *A. butzleri* and *A. cryaerophilus* was 2.38 Mbp. All sequenced genomes were complete and circular. Several isolates contained multiple contigs attributable to plasmids. However, no known replicon types were identified. Plasmids were found in 8/27 sequenced *A. butzleri* and in 6/6 *A. cryaerophilus*. None of the isolates shared identical plasmids. However, the plasmid gene sharing network indicated active gene exchange within and between *A. butzleri* and *A. cryaerophilus* plasmids (Fig. 3).

A phylogenetic tree based on 16S rRNA gene sequences of the recovered isolates and *Arcobacter* ASVs confirmed that the isolates clustered well within the *Arcobacter* diversity that was observed with amplicon sequencing (Fig. S3). Interestingly, three *A. cryaerophilus* isolates (ENC4, ENC5, and ENC6) showed intragenomic variation among the 16S rRNA copies in their genome, with two to six nucleotide differences observed. In contrast, almost all 16S rRNA copies in the *A. butzleri* genomes were identical (only AEC8 had one nucleotide difference in one 16S rRNA copy). Phylogenetic analysis including all ASVs and 16S rRNA gene copies extracted from the genomes showed that distinct intragenomic variants of *A. cryaerophilus* clustered with separate ASVs (Fig. S3), indicating that intragenomic 16S rRNA heterogeneity contributed to the observed *A. cryaerophilus* ASV diversity.

To achieve higher phylogenetic resolution, whole-genome phylogenetic analysis was performed and revealed that all six *A. cryaerophilus* isolates belonged to subclade IV when compared with representative genomes for each subclade (59) (Fig. S4).

### Genetic background of antibiotic resistance

**Fig 4:**
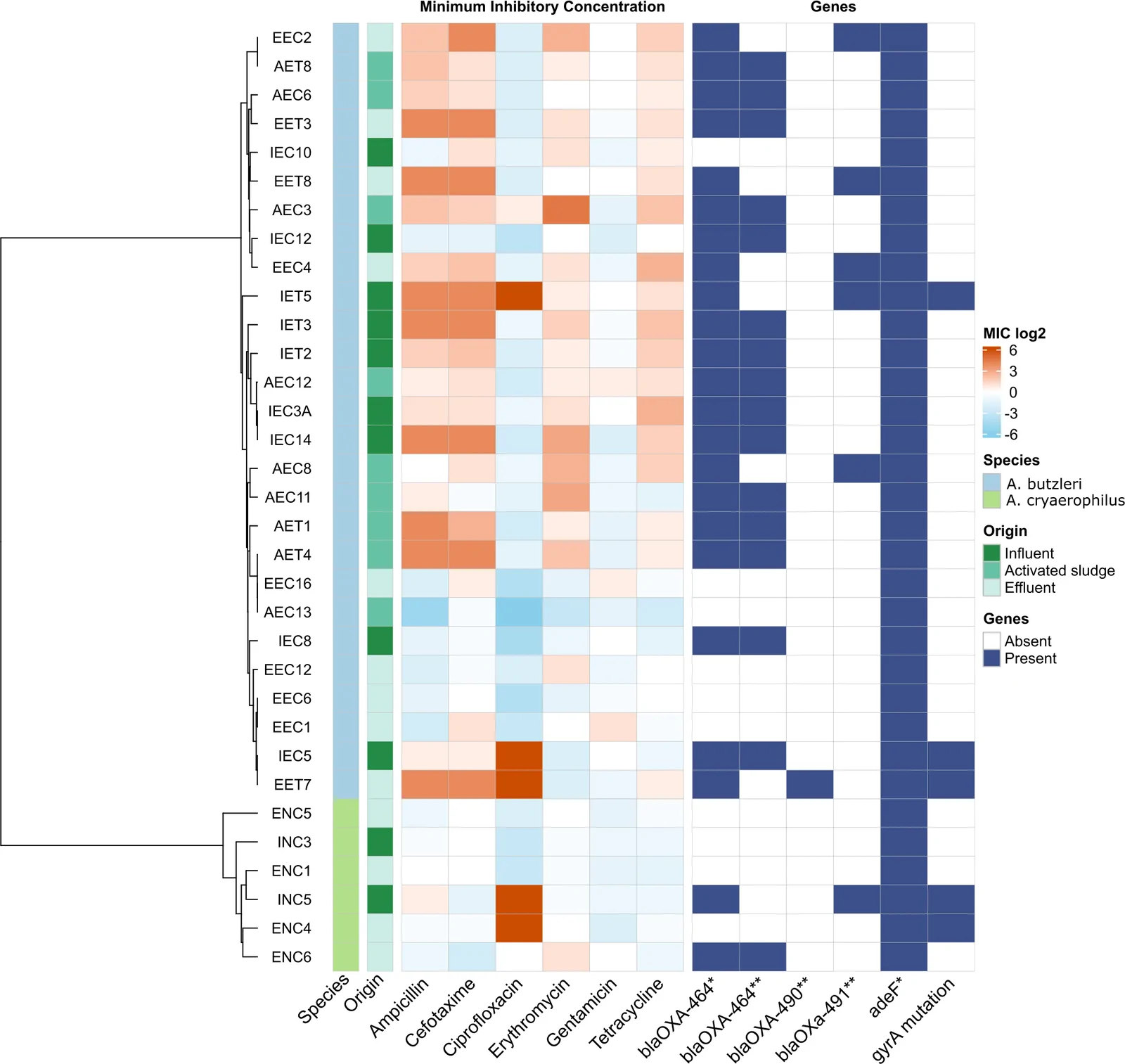
Antibiotic resistance profile of 33 sequenced *Arcobacter* isolates showing log_2_ transformed minimum inhibitory concentrations (MICs) in relation to epidemiological cutoff (ECOFF for *C. jejuni*) values for each studied antibiotic. Since no official ECOFF value for cefotaxime is available for *C. jejuni*, a hypothetical threshold value of 16 μg/mL was applied for visualization. Red indicates MICs above the ECOFF (non-wild phenotype), and blue indicates MICs at or below the ECOFF (wild phenotype). Isolates are ordered according to their phylogenetic relationship inferred from single-copy core genes. For all displayed genetic determinants of antibiotic resistance, blue indicates gene presence and white indicates gene absence. Genes detected in the CARD database are marked with a single asterisk (*), and those detected in ResFinder are marked with a double asterisk (**).

Isolates with closer genetic relatedness tended to share similar antibiotic resistance profiles (Fig. 4). According to the CARD database, only one β-lactamase, *bla*_OXA-464_, was detected in 23 isolates, while ResFinder additionally identified *bla*_OXA-464_ (16 isolates), *bla*_OXA-490_ (1 isolate), and *bla*_OXA-491_ (6 isolates) (Tables S4 and S5). These β-lactamases were associated with isolates exhibiting high MICs for ampicillin and cefotaxime, although some isolates with low MICs also carried these genes. All *bla*_OXA_ genes were chromosomally located and showed no evidence of mobility based on analysis of the genetic context.

In addition, a point mutation (Thr-85-Ile) in the *gyrA* gene was found in isolates with elevated MICs for ciprofloxacin (>32 µg/mL). The *adeF* gene, encoding a component of the resistance–nodulation–cell division (RND) efflux pump, was detected in all sequenced isolates, indicating a strong efflux potential.

### Heavy metal and biocide resistance

**Fig 5:**
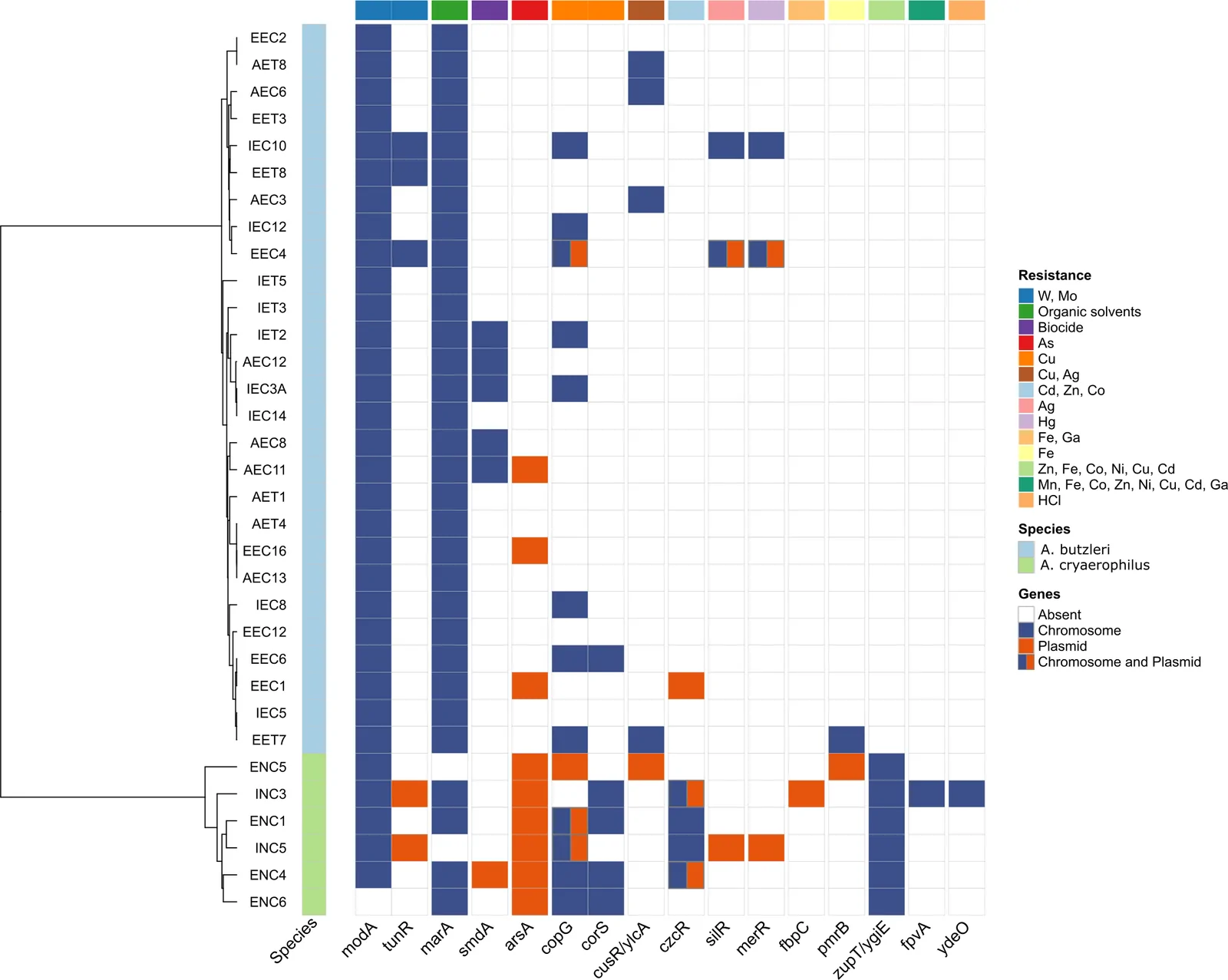
Presence and genomic location (chromosome, plasmid, or both) of genes associated with resistance to heavy metals, organic solvents, biocides, and hydrochloric acid in 33 sequenced *Arcobacter* isolates screened against the BacMet database. Elemental abbreviations used correspond to chemical element symbols.

Screening against the BacMet database identified resistance-associated genes for heavy metals (*n* = 11), organic solvents (*n* = 1), biocides (*n* = 1), and hydrochloric acid (*n* = 1) (Fig. 5 and Table S6). The most prevalent gene was *modA*, conferring resistance to tungsten and molybdenum, detected in 32/33 isolates. The transcriptional regulator *marA*, associated with organic solvent tolerance and multidrug resistance, was present in 31/33 isolates. On average, *A. cryaerophilus* harbored more resistance genes per isolate (7.33 ± 1.75) compared to *A. butzleri* (3.07 ± 1.17). However, the markedly lower number of *A. cryaerophilus* isolates compared with *A. butzleri* limits the robustness of species-level comparisons. The isolate with the highest number of resistance genes was INC3 (*n* = 10).

Genes *modA* and *marA* were consistently located on the chromosome, whereas *arsA* was exclusively plasmid-borne. Other resistance genes showed variable localization, being detected either on the chromosome or on plasmids, depending on the isolate. Notably, *copG*, *czcR*, *silR*, and *merR* were in some cases present in two copies within the same isolate, with one copy located on the chromosome and another on a plasmid. In these cases, it was evident that the gene had mobilized from chromosome to plasmid or vice versa. Resistance genes on plasmids were detected in all 6 *A. cryaerophilus* isolates but only in 4/27 *A. butzleri* isolates. Among them, the *A. cryaerophilus* isolate INC5 carried the highest plasmid gene content (*n* = 5) (Fig. 5). The plasmid from the *A. butzleri* isolate EEC1 harbored similar genes as in *A. cryaerophilus* plasmids, which is also seen in the plasmid gene-sharing network (Fig. 3).

## DISCUSSION

This study represents a systematic investigation of *Arcobacter* diversity, antibiotic resistance, and genetic traits in a Finnish wastewater treatment plant using amplicon sequencing combined with isolate-based genomic and phenotypic antibiotic susceptibility testing, revealing high abundance in raw sewage, persistence through treatment, and the presence of diverse resistance determinants.

*Arcobacter* was abundant in raw sewage (41.8%) and was effectively reduced during wastewater treatment (1.9%). In a study from the same WWTP, Hultman et al. (15) reported a high relative abundance (39%–43%) of *Campylobacterales*, which could correspond to the *Arcobacter* genus in influents and a significant reduction in effluents (6.68%). In a recent study from Italy, the abundance of arcobacters was consistent across the three WWTPs, with 22% in influents and a reduction to 12% in effluents (60). In contrast, Kristensen et al. (61) reported *Arcobacter* among the top 10 taxa in 14 Danish WWTPs, with varying levels of removal efficiency. Such differences may reflect site-specific factors, including treatment technology, plant size, hydraulic retention time, or operational conditions that favor survival of certain species.

*A. cryaerophilus* and *A. suis* were the most represented *Arcobacter* species in wastewater, whereas *A. butzleri* and *A. cibarius* had lower proportions. In addition, 96 ASVs could not be classified to the species level, which may indicate uncharacterized *Arcobacter* diversity not yet recorded in reference databases. Kristensen et al. (61) recorded 15 *Arcobacter* species in the metagenomes from the 14 investigated WWTPs. However, only three full-length 16S rRNA genes were assembled that corresponded to *A. cryaerophilus* and *A. suis*, with *A. cryaerophilus* being the most abundant among the metagenomes, which is in accordance with the present study. During wastewater treatment, there was a notable increase in *A. cryaerophilus* proportions within the *Arcobacter* genus in effluents, which indicates that some species can withstand or even proliferate under treatment conditions. Since most published studies rely on short-read sequencing and report only genus-level data, direct comparison of species-level dynamics across WWTPs remains limited.

There are currently numerous variations of isolation protocols for the recovery of *Arcobacter* spp. (11). The protocol employed in this study successfully yielded 81 isolates. Combined enrichment and filtration strategies using 0.45 µm filters have been shown to be effective for recovering *Arcobacter* from complex wastewater matrices (61). Similarly, enrichment in our study significantly improved *Arcobacter* recovery. However, it also introduced a bias toward faster-growing species such as *A. butzleri*, potentially leading to underrepresentation of slower-growing taxa. This observation is consistent with Levican et al. (62), who reported that direct plating without enrichment resulted in greater species diversity.

Passive filtration is commonly used for *Arcobacter* recovery (11). In contrast, we applied syringe filtration through 0.45 µm filters after enrichment to reduce background flora, which proved to be time efficient. Filtration was effective only when target bacteria were sufficiently abundant, as no growth was observed in non-enriched samples. Moreover, syringe filtration may exclude *Arcobacter* cells associated with aggregates, potentially limiting overall recovery. The addition of tetracycline to the CAT supplement enhanced the recovery of isolates with non-wild phenotypes for multiple antibiotics, suggesting that CAT alone may not provide sufficient selectivity. When syringe filtration is not applied, additional antibiotic supplementation may be necessary to further suppress background flora (11). Future modifications of the cultivation protocol could focus on improving both selectivity and the recovery of a broader species diversity.

The isolates recovered in this study clustered within the broader *Arcobacter* diversity detected by amplicon sequencing but reflected the bias of culture-based approaches toward non-fastidious species. Whole genome-based pangenomic analysis further demonstrated that all six *A. cryaerophilus* isolates grouped within subclade IV when compared with representative genomes from four established phylogenomic subclades (59). This finding is notable, given that *A. cryaerophilus* has been described as a genetically heterogeneous species complex comprising multiple distinct lineages (2). The placement of our isolates within a single subclade indicates that they represent a genomically coherent lineage rather than spanning the full diversity of the species complex. Moreover, detailed inspection of the 16S rRNA gene sequences revealed intragenomic variation among the five 16S rRNA gene copies in some *A. cryaerophilus* isolates. Such intragenomic heterogeneity can inflate ASV counts and potentially lead to overestimation of species diversity in amplicon-based studies (63). Together, these findings highlight both the taxonomic complexity within *A. cryaerophilus* and the limitations of relying solely on 16S rRNA gene-based approaches.

Currently, there are no official guidelines for AST of *Arcobacter* spp. Studies have employed diverse growth media, inoculum sizes, oxygen conditions, incubation temperatures, and durations for AST. MIC values were often compared with clinical breakpoints established for *Enterobacterales*, *Streptococcus* spp., *Campylobacter*, or other unrelated species according to CLSI or EUCAST guidelines (17, 18, 21, 64–67). Consequently, AST studies tended to show inconsistent results, highlighting the difficulties in comparison between studies and the urgent need for standardized methods. In the present study, MIC gradient strips were used, which is consistent with several previous *Arcobacter* susceptibility studies (17, 20, 66). Although gradient diffusion methods provide quantitative MIC measurements, they may produce MIC values that differ from the EUCAST reference broth microdilution method, particularly for fastidious organisms. ECOFF values were used to interpret antibiotic susceptibility in the absence of *Arcobacter*-specific breakpoints. ECOFFs are derived from phenotypic measurements and define the upper limit of the wild-type population distribution, serving as an initial step in the establishment of clinical breakpoints. They are also used for antimicrobial resistance surveillance when clinical breakpoints are unavailable, insufficiently sensitive, or vary between systems (68). Because no ECOFFs exist for *Arcobacter*, those for *Campylobacter jejuni* were applied as in Jehanne et al. (20).

Antibiotic susceptibility testing revealed high proportions of non-wild-type isolates for ampicillin, cefotaxime, erythromycin, and tetracycline, whereas the proportion of non-wild types for ciprofloxacin and gentamicin was low. These findings are consistent with previous studies that applied similar testing conditions and interpretation criteria to clinical and environmental isolates (17, 20, 21).

Elevated MIC values for ampicillin in the investigated isolates appear to be mediated by chromosomally encoded β-lactamases *bla*_OXA-464_, *bla*_OXA-490_, and *bla*_OXA-491_, which have previously been reported in *Arcobacter* spp. (20, 69). Variability in the expression of these genes may contribute to the observed differences in MIC values among isolates. The cefotaxime MIC profile resembled that of ampicillin. However, while *bla*_OXA-464_ is known to confer resistance to penicillins, no evidence currently links it to third-generation cephalosporins. This suggests that elevated MIC values for cefotaxime in the present isolates could be mediated by alternative mechanisms such as expression of efflux pumps (70).

Elevated ciprofloxacin MIC values were associated with a point mutation (Thr-85-Ile) in the *gyrA* gene, a well-known resistance mechanism in *A. butzleri* (71). No conclusive mechanisms were identified for elevated MIC values for erythromycin, gentamicin, and tetracycline, such as the presence of *ereA*, *macB*, *tetW*, *tetA*, *tetO*, or *aadA2* genes previously described in the literature (72). According to a previous study, low-level erythromycin resistance in *A. butzleri* is typically associated with increased efflux activity, whereas high-level resistance results from a combination of increased efflux and reduced antibiotic binding due to mutations in the L4 and L22 ribosomal proteins (73). In the present study, no mutations were detected in L4 or L22.

The presence of the *adeF* gene, encoding a component of the RND efflux pump, in all sequenced isolates suggests a strong efflux potential that may contribute to slightly elevated MIC values across multiple antibiotic classes such as tetracyclines and fluoroquinolones (72). In addition, we have detected a multitude of putative uncharacterized efflux systems in the genomic annotations that could play a role in multidrug resistance.

In the context of One Health, the antibiotic resistance genes identified in this study are not restricted to *Arcobacter* from wastewater. The *gyrA* Thr-85-Ile mutation has been reported in isolates from water poultry samples (67) as well as in human clinical isolates (66). β-Lactamases of the OXA family have been detected in water, seafood, and poultry (72) and also in human isolates (20). The efflux gene *adeF* has been observed in cow milk isolates (72). Together, these findings illustrate the circulation of antibiotic resistance determinants across clinical, food, and environmental reservoirs.

In our sequenced *Arcobacter* isolates, we have not found antibiotic resistance genes that were previously detected with epicPCR (14–16). This could be explained by these genes being absent or rare within the studied *Arcobacte*r population, making them difficult to detect using cultivation-based methods. The similarity of antibiotic resistance profiles among genetically related isolates, combined with the absence of mobile resistance determinants, indicates that antibiotic resistance in studied *Arcobacter* isolates is predominantly chromosomal. The frequent occurrence of *blaOXA* genes in *Arcobacter* raises the question of whether these genes should be considered intrinsic. However, because they were not detected in all isolates in our collection (23/33 carried a *blaOXA* gene), they cannot be regarded as part of the core genome. It is possible that these genes were acquired in an ancestral lineage and subsequently integrated into the chromosome, followed by loss in some strains. Nevertheless, our data do not support classifying *blaOXA* genes as universally intrinsic. Instead, they appear to represent common but not ubiquitous β-lactam resistance determinants within this genus.

A broad arsenal of resistance-associated genes to heavy metals, biocides, and organic solvents in the investigated isolates indicates strong adaptation to the chemically complex environment of wastewater treatment plants. The high prevalence of *modA* and *marA* across nearly all isolates demonstrates that metal resistance and organic solvent tolerance are widespread in this genus. Furthermore, *marA* is a well-known transcriptional regulator in enteric bacteria that has been reported to modulate efflux pump and porin expression as well as genes required for lipid trafficking and DNA repair, thus potentially contributing to multidrug resistance (74, 75). The higher number of resistance genes in *A. cryaerophilus* compared to *A. butzleri* suggests that *A. cryaerophilus* is particularly well adapted to survive in polluted environments, which may partly explain its high relative abundance in sewage. The detection of resistance genes on chromosomes, plasmids, or both highlights a mixed strategy of vertical inheritance and potential horizontal transfer. Notably, the high content of plasmid-borne resistance genes in *A. cryaerophilus* suggests that this species may act as an important reservoir and potential donor of non-antibiotic resistance determinants that promote survival within wastewater microbial communities.

In the literature, whole-genome analyses of *Arcobacter* spp. consistently report a broad array of putative antimicrobial and heavy-metal resistance determinants, suggesting genetic potential for multidrug resistance, metal tolerance, and co-selection (64, 69, 76). However, most evidence derives from *in silico* annotation and comparative genomics, while experimental validation of gene expression and genotype–phenotype correlations remain limited. These observations, together with the absence of standardized antibiotic susceptibility testing guidelines for *Arcobacter*, highlight critical gaps that warrant further investigation. This study employed a cross-sectional sampling design, in which wastewater and activated sludge were collected at a single time point, providing a robust snapshot of *Arcobacter* occurrence, diversity, and antimicrobial resistance patterns in the investigated systems. While this approach does not capture temporal variability, the comprehensive characterization of isolates and MIC profiles supports the validity of our findings within the studied time and location. Future studies incorporating longitudinal and geographically broader sampling would be valuable to assess temporal dynamics and generalize these results to other wastewater treatment plants.

### Conclusion

This study provides a systematic investigation of *Arcobacter* spp. in a wastewater treatment system, revealing species-level diversity, intragenomic 16S rRNA variability, and widespread antibiotic resistance predominantly mediated by chromosomally encoded determinants. Our findings also highlight genetic traits, such as genes for heavy metal, biocide, and organic solvent resistance, that may support the persistence of *Arcobacter* in anthropogenically influenced environments and establish a valuable baseline for future functional and ecological research.

## Data Availability

All raw reads and assembled genomes are available from the Sequence Read Archive and European Nucleotide Archive under the project accession PRJEB98879. All annotated genomes have been uploaded to Zenodo (DOI is 10.5281/zenodo.18010346).

## SUPPLEMENTAL MATERIAL

Fig. S1 spectrum.03799-25-s0001.pdf Relative abundance of the 20 most abundant genera in influent, activated sludge, and effluent samples from WWTP Viikinmäki. Principal coordinate analysis (PCoA) was based on Aitchison distances.

Fig. S2 spectrum.03799-25-s0002.pdf Principal coordinate analysis (PCoA) based on Euclidean distances of log₂-transformed MIC values for Arcobacter isolates (*A. butzleri n* = 75; *A. cryaerophilus n* = 6).

Fig. S3 spectrum.03799-25-s0003.pdf Phylogenetic tree including all full-length amplicon sequence variants (ASVs, red) assigned to Arcobacter and unique 16S rRNA gene sequences from each sequenced isolate (blue).

Fig. S4 spectrum.03799-25-s0004.pdf Phylogenetic tree including all six Arcobacter cryaerophilus isolates and representative genomes from four established phylogenomic subclades.

Tables S1–S6 spectrum.03799-25-s0005.xlsx Diversity and antibiotic resistance of Arcobacter species in a wastewater treatment system.

Supplemental Figure Legends spectrum.03799-25-s0006.docx Legends for Figures S1–S4.

ASM does not own the copyrights to Supplemental Material that may be linked to, or accessed through, an article. The authors have granted ASM a non-exclusive, world-wide license to publish the Supplemental Material files. Please contact the corresponding author directly for reuse.
