# Supplementary material for: Emerging food- and waterborne pathogen Arcobacter in wastewater: diversity and antibiotic resistance
Source: Microbiol Spectr. 2026 Sep 17;14(10):e03799-25. doi: 10.1128/spectrum.03799-25 (PMC13689115; doi:10.1128/spectrum.03799-25)
Supplement: Supplementary file 1 [file spectrum.03799-25-s0001.pdf]

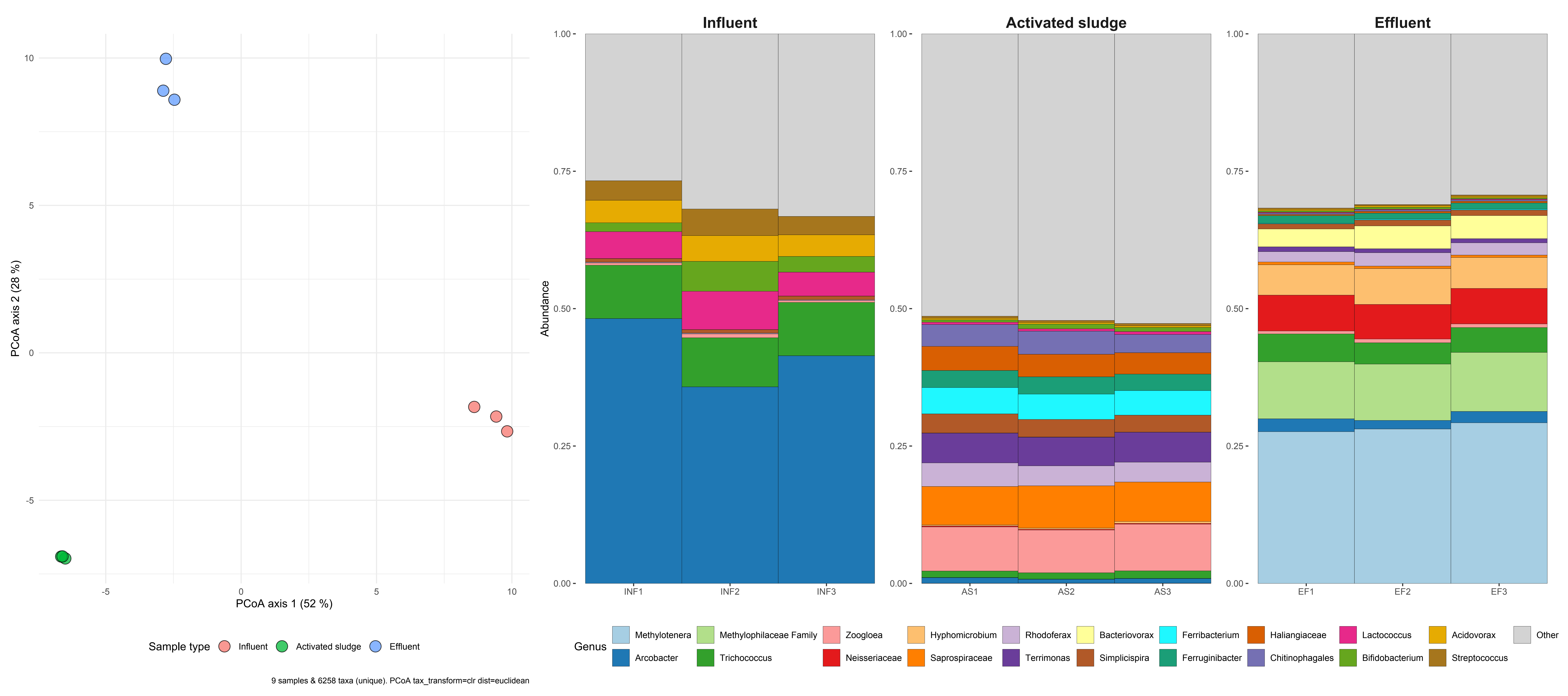

PCoA axis 2 (28 %)
PCoA axis 1 (52 %)
Sample type
Influent
Activated sludge
Effluent
9 samples & 6258 taxa (unique). PCoA tax_transform=clr dist=euclidean
Influent
Activated sludge
Effluent
Abundance
INF1
INF2
INF3
AS1
AS2
AS3
EF1
EF2
EF3
Genus
Methylotenera
Arcobacter
Methylophilaceae Family
Trichococcus
Zoogloea
Neisseriaceae
Hyphomicrobium
Saprospiraceae
Rhodoferax
Terrimonas
Bacteriovorax
Simplicispira
Ferribacterium
Ferruginibacter
Haliangiaceae
Chitinophagales
Lactococcus
Bifidobacterium
Acidovorax
Streptococcus
Other
