# Supplementary material for: Emerging food- and waterborne pathogen Arcobacter in wastewater: diversity and antibiotic resistance
Source: Microbiol Spectr. 2026 Sep 17;14(10):e03799-25. doi: 10.1128/spectrum.03799-25 (PMC13689115; doi:10.1128/spectrum.03799-25)
Supplement: Supplementary file 2 [file spectrum.03799-25-s0002.pdf]

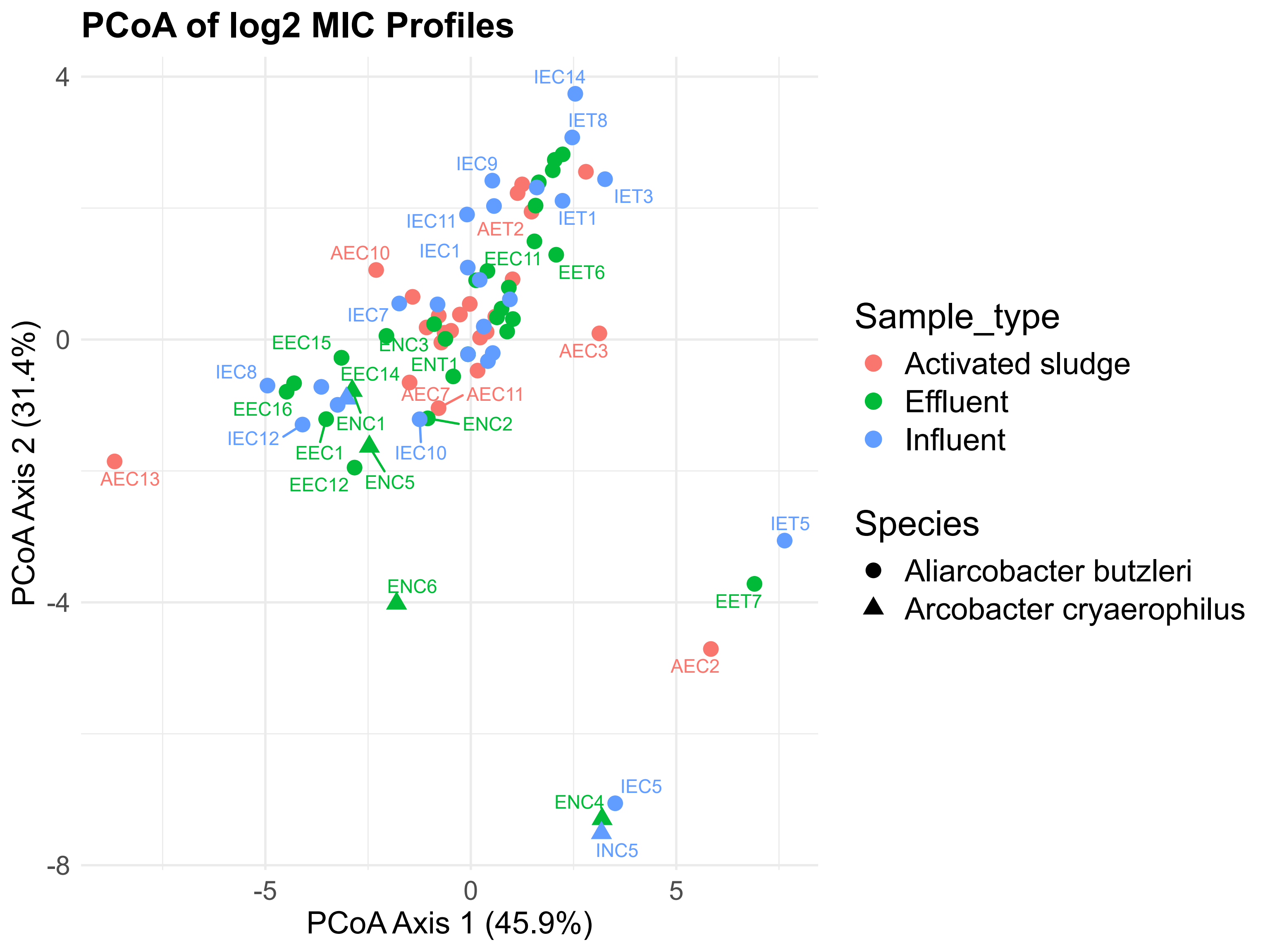

PCoA of log2 MIC Profiles
PCoA Axis 1 (45.9%)
PCoA Axis 2 (31.4%)
4
0
-4
-8
-5
0
5
IEC14
IET8
IEC9
IET3
IET1
IEC11
AET2
AEC10
IEC1
EEC11
EET6
IEC7
EEC15
ENC3
AEC3
ENT1
IEC8
EEC14
AEC7
AEC11
EEC16
ENC1
ENC2
IEC12
EEC1
IEC10
AEC13
EEC12
ENC5
IET5
ENC6
EET7
AEC2
IEC5
ENC4
INC5
Sample_type
Activated sludge
Effluent
Influent
Species
Aliarcobacter butzleri
Arcobacter cryaerophilus
