# Supplementary material for: Emerging food- and waterborne pathogen Arcobacter in wastewater: diversity and antibiotic resistance
Source: Microbiol Spectr. 2026 Sep 17;14(10):e03799-25. doi: 10.1128/spectrum.03799-25 (PMC13689115; doi:10.1128/spectrum.03799-25)
Supplement: Supplementary file 3 [file spectrum.03799-25-s0003.pdf]

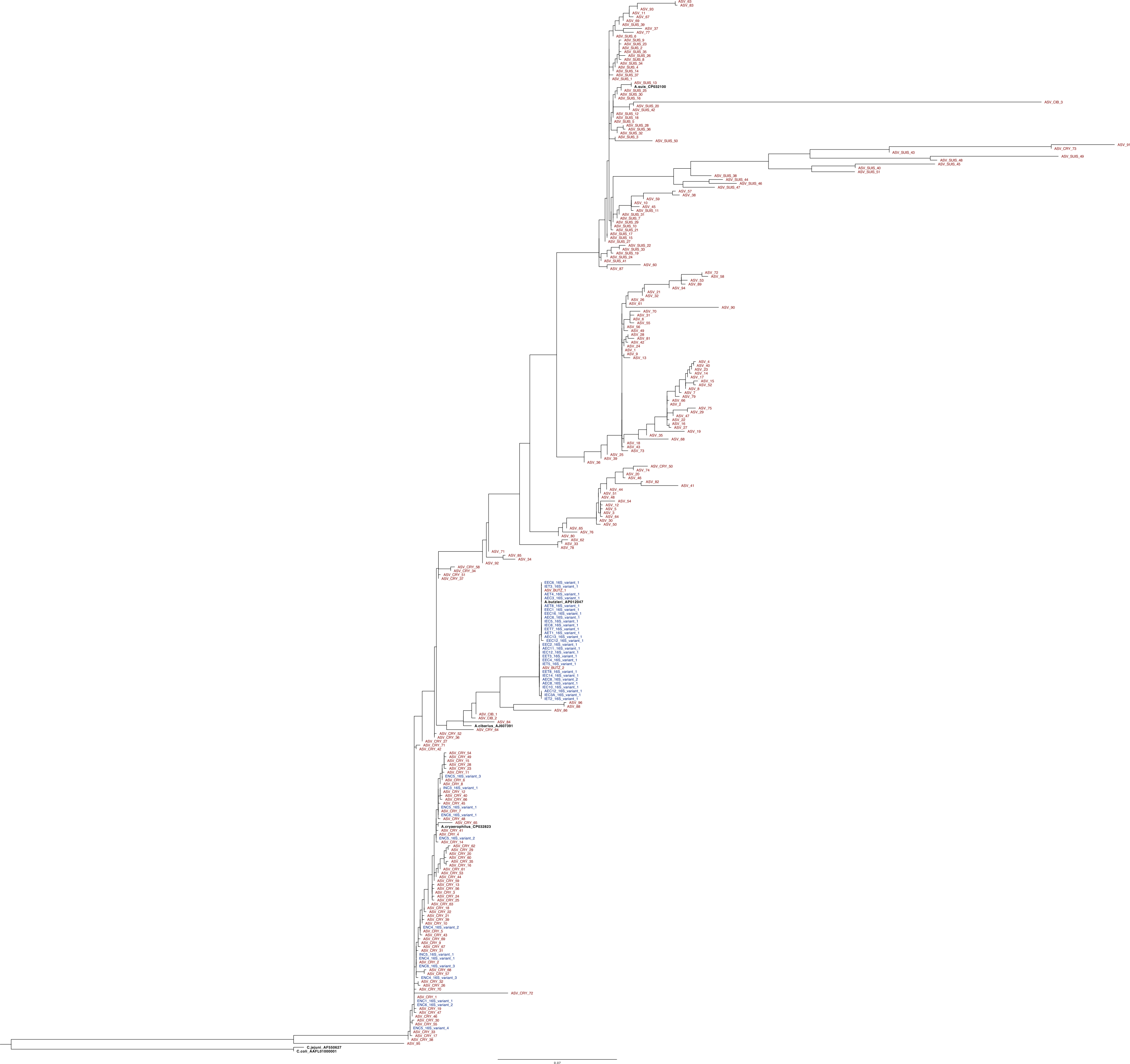
ASV_63
ASV_83
ASV_93
ASV_11
ASV_67
ASV_69
ASV_39
ASV_37
ASV_77
ASV_SUIS_6
ASV_SUIS_9
ASV_SUIS_23
ASV_SUIS_2
ASV_SUIS_35
ASV_SUIS_26
ASV_SUIS_8
ASV_SUIS_34
ASV_SUIS_4
ASV_SUIS_14
ASV_SUIS_37
ASV_SUIS_1
ASV_SUIS_13
A.suis_CP032100
ASV_SUIS_25
ASV_SUIS_30
ASV_SUIS_16
ASV_CIB_3
ASV_SUIS_20
ASV_SUIS_42
ASV_SUIS_12
ASV_SUIS_18
ASV_SUIS_5
ASV_SUIS_28
ASV_SUIS_36
ASV_SUIS_32
ASV_SUIS_3
ASV_SUIS_50
ASV_91
ASV_CRY_73
ASV_SUIS_43
ASV_SUIS_49
ASV_SUIS_48
ASV_SUIS_45
ASV_SUIS_40
ASV_SUIS_51
ASV_SUIS_38
ASV_SUIS_44
ASV_SUIS_46
ASV_SUIS_47
ASV_57
ASV_38
ASV_59
ASV_10
ASV_45
ASV_SUIS_11
ASV_SUIS_31
ASV_SUIS_7
ASV_SUIS_29
ASV_SUIS_10
ASV_SUIS_21
ASV_SUIS_17
ASV_SUIS_15
ASV_SUIS_27
ASV_SUIS_22
ASV_SUIS_33
ASV_SUIS_19
ASV_SUIS_24
ASV_SUIS_41
ASV_60
ASV_87
ASV_72
ASV_58
ASV_53
ASV_89
ASV_94
ASV_21
ASV_32
ASV_26
ASV_61
ASV_90
ASV_70
ASV_31
ASV_6
ASV_55
ASV_56
ASV_49
ASV_28
ASV_81
ASV_42
ASV_24
ASV_1
ASV_9
ASV_13
ASV_4
ASV_40
ASV_23
ASV_14
ASV_17
ASV_15
ASV_52
ASV_8
ASV_7
ASV_79
ASV_66
ASV_2
ASV_75
ASV_29
ASV_47
ASV_22
ASV_16
ASV_27
ASV_19
ASV_35
ASV_68
ASV_18
ASV_43
ASV_73
ASV_25
ASV_39
ASV_36
ASV_CRY_50
ASV_74
ASV_20
ASV_46
ASV_82
ASV_41
ASV_44
ASV_51
ASV_48
ASV_54
ASV_12
ASV_5
ASV_3
ASV_64
ASV_30
ASV_50
ASV_65
ASV_76
ASV_80
ASV_62
ASV_33
ASV_78
ASV_71
ASV_85
ASV_34
ASV_92
ASV_CRY_58
ASV_CRY_34
ASV_CRY_51
ASV_CRY_37
EEC6_16S_variant_1
IET3_16S_variant_1
ASV_BUTZ_1
AET4_16S_variant_1
AEC3_16S_variant_1
A.butzleri_AP012047
AET8_16S_variant_1
EEC1_16S_variant_1
EEC16_16S_variant_1
AEC6_16S_variant_1
IEC5_16S_variant_1
IEC9_16S_variant_1
EET7_16S_variant_1
AET1_16S_variant_1
AEC13_16S_variant_1
EEC12_16S_variant_1
EEC2_16S_variant_1
AEC11_16S_variant_1
IEC12_16S_variant_1
EET3_16S_variant_1
EEC4_16S_variant_1
IET5_16S_variant_1
ASV_BUTZ_2
EET8_16S_variant_1
IEC14_16S_variant_1
AEC8_16S_variant_2
AEC8_16S_variant_1
IEC10_16S_variant_1
AEC12_16S_variant_1
IEC3A_16S_variant_1
IET2_16S_variant_1
ASV_96
ASV_88
ASV_86
ASV_CIB_1
ASV_CIB_2
ASV_84
A.cibarius_AJ607391
ASV_CRY_64
ASV_CRY_52
ASV_CRY_36
ASV_CRY_27
ASV_CRY_71
ASV_CRY_42
ASV_CRY_54
ASV_CRY_49
ASV_CRY_15
ASV_CRY_28
ASV_CRY_23
ASV_CRY_11
ENC5_16S_variant_3
ASV_CRY_6
ASV_CRY_8
INC3_16S_variant_1
ASV_CRY_12
ASV_CRY_40
ASV_CRY_66
ASV_CRY_45
ENC5_16S_variant_1
ASV_CRY_7
ENC6_16S_variant_1
ASV_CRY_48
ASV_CRY_65
A.cryaerophilus_CP032823
ASV_CRY_41
ASV_CRY_4
ENC5_16S_variant_2
ASV_CRY_14
ASV_CRY_62
ASV_CRY_29
ASV_CRY_20
ASV_CRY_60
ASV_CRY_35
ASV_CRY_16
ASV_CRY_61
ASV_CRY_53
ASV_CRY_44
ASV_CRY_59
ASV_CRY_13
ASV_CRY_56
ASV_CRY_3
ASV_CRY_24
ASV_CRY_25
ASV_CRY_63
ASV_CRY_18
ASV_CRY_22
ASV_CRY_21
ASV_CRY_39
ASV_CRY_10
ENC4_16S_variant_2
ASV_CRY_5
ASV_CRY_43
ASV_CRY_69
ASV_CRY_9
ASV_CRY_67
ASV_CRY_31
INC5_16S_variant_1
ENC4_16S_variant_1
ASV_CRY_2
ENC6_16S_variant_3
ASV_CRY_68
ASV_CRY_57
ENC4_16S_variant_3
ASV_CRY_32
ASV_CRY_26
ASV_CRY_70
ASV_CRY_72
ASV_CRY_1
ENC1_16S_variant_1
ENC6_16S_variant_2
ASV_CRY_19
ASV_CRY_47
ASV_CRY_46
ASV_CRY_30
ASV_CRY_55
ENC5_16S_variant_4
ASV_CRY_33
ASV_CRY_17
ASV_CRY_38
ASV_95
C.jejuni_AF550627
C.coli_AAFL01000001
0.07
