# Supplementary material for: Emerging food- and waterborne pathogen Arcobacter in wastewater: diversity and antibiotic resistance
Source: Microbiol Spectr. 2026 Sep 17;14(10):e03799-25. doi: 10.1128/spectrum.03799-25 (PMC13689115; doi:10.1128/spectrum.03799-25)
Supplement: Supplementary file 4 [file spectrum.03799-25-s0004.pdf]

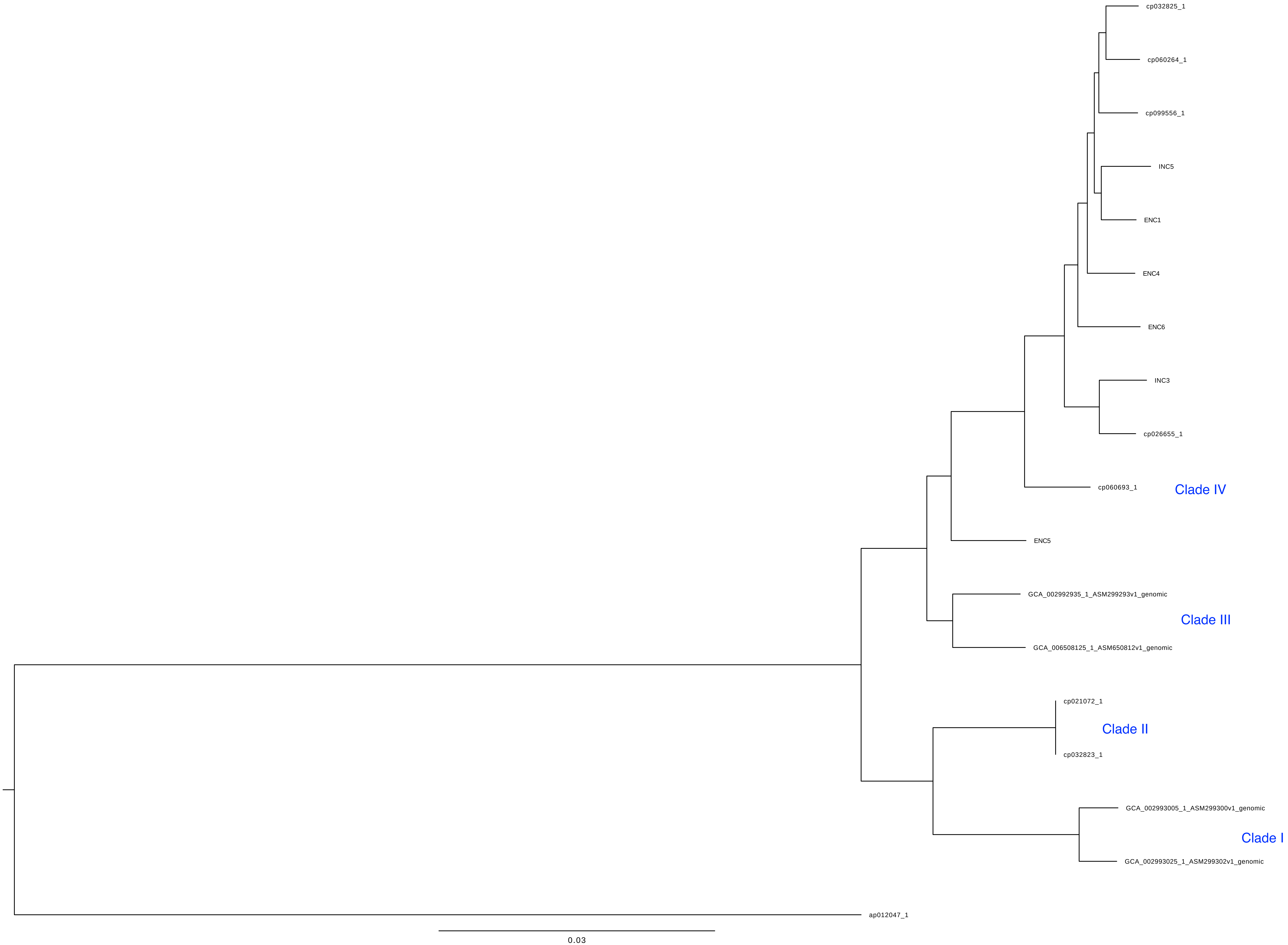
cp032825_1
cp060264_1
cp099556_1
INC5
ENC1
ENC4
ENC6
INC3
cp026655_1
cp060693_1
Clade IV
ENC5
GCA_002992935_1_ASM299293v1_genomic
Clade III
GCA_006508125_1_ASM650812v1_genomic
cp021072_1
Clade II
cp032823_1
GCA_002993005_1_ASM299300v1_genomic
Clade I
GCA_002993025_1_ASM299302v1_genomic
ap012047_1
0.03
