# Supplementary material for: Emerging food- and waterborne pathogen Arcobacter in wastewater: diversity and antibiotic resistance
Source: Microbiol Spectr. 2026 Sep 17;14(10):e03799-25. doi: 10.1128/spectrum.03799-25 (PMC13689115; doi:10.1128/spectrum.03799-25)
Supplement: Supplementary file 5 [file spectrum.03799-25-s0006.docx]

**Supplementary figure 1**

Relative abundance of the 20 most abundant genera in influent, activated sludge, and effluent samples from WWTP Viikinmäki. Principal coordinate analysis (PCoA) was based on Aitchison distances. Bacterial community composition showed clear clustering according to sample type.

**Supplementary figure 2**

Principal coordinate analysis (PCoA) based on Euclidean distances of log₂-transformed MIC values for *Arcobacter* isolates (*A. butzleri* n = 75; *A. cryaerophilus* n = 6). No clear clustering according to sample type was observed, which was confirmed by PERMANOVA analysis. However, PERMANOVA indicated that species explained a small but statistically significant proportion of the variation in MIC profiles. The imbalance in sample sizes limits interpretation of species-level differences, and additional *A. cryaerophilus* isolates would improve statistical robustness.

**Supplementary figure 3**

Phylogenetic tree including all full-length amplicon sequence variants (ASVs; red) assigned to *Arcobacter* and unique 16S rRNA gene sequences from each sequenced isolate (blue). Reference species are shown in black. In total, 225 distinct ASVs were assigned to *Arcobacter*. Species designations are as follows: *A. suis* ASV_SUIS_1–51; *A. butzleri* ASV_BUTZ_1–2; *A. cibarius* ASV_CIB_1–3; and *A. cryaerophilus* ASV_CRY_1–73. In addition, 96 ASVs could not be confidently assigned to species level and were designated ASV_1–96. Phylogenetic analysis showed that the sequenced isolates clustered within the *Arcobacter* diversity detected by amplicon sequencing.

**Supplementary figure 4.**

Phylogenetic tree including all six *Arcobacter cryaerophilus* isolates and representative genomes from four established phylogenomic subclades. Whole-genome phylogenetic analysis was performed to achieve higher phylogenetic resolution and showed that all six isolates belonged to subclade IV.
